# Positive end-expiratory pressure management in patients with severe ARDS: implications of prone positioning and extracorporeal membrane oxygenation

**DOI:** 10.1186/s13054-024-05059-y

**Published:** 2024-08-26

**Authors:** Christoph Boesing, Patricia R. M. Rocco, Thomas Luecke, Joerg Krebs

**Affiliations:** 1 Department of Anesthesiology and Critical Care Medicine, University Medical Center Mannheim, Medical Faculty Mannheim, University of Heidelberg, Theodor-Kutzer-Ufer 1-3, 68167 Mannheim, Germany; 2 Laboratory of Pulmonary Investigation, Carlos Chagas Filho Institute of Biophysics, Federal University of Rio de Janeiro, Centro de Ciências da Saúde, Avenida Carlos Chagas Filho, 373, Bloco G-014, Ilha do Fundão, Rio de Janeiro, Brazil

**Keywords:** Acute respiratory distress syndrome, Positive end-expiratory pressure, Respiratory mechanics, Prone positioning, Extracorporeal membrane oxygenation, Ventilator-induced lung injury, Lung protective ventilation

## Abstract

The optimal strategy for positive end-expiratory pressure (PEEP) titration in the management of severe acute respiratory distress syndrome (ARDS) patients remains unclear. Current guidelines emphasize the importance of a careful risk–benefit assessment for PEEP titration in terms of cardiopulmonary function in these patients. Over the last few decades, the primary goal of PEEP usage has shifted from merely improving oxygenation to emphasizing lung protection, with a growing focus on the individual pattern of lung injury, lung and chest wall mechanics, and the hemodynamic consequences of PEEP. In moderate-to-severe ARDS patients, prone positioning (PP) is recommended as part of a lung protective ventilation strategy to reduce mortality. However, the physiologic changes in respiratory mechanics and hemodynamics during PP may require careful re-assessment of the ventilation strategy, including PEEP. For the most severe ARDS patients with refractory gas exchange impairment, where lung protective ventilation is not possible, veno-venous extracorporeal membrane oxygenation (V-V ECMO) facilitates gas exchange and allows for a “lung rest” strategy using “ultraprotective” ventilation. Consequently, the importance of lung recruitment to improve oxygenation and homogenize ventilation with adequate PEEP may differ in severe ARDS patients treated with V-V ECMO compared to those managed conservatively. This review discusses PEEP management in severe ARDS patients and the implications of management with PP or V-V ECMO with respect to respiratory mechanics and hemodynamic function.

## Background

Positive end-expiratory pressure (PEEP) has been a cornerstone in the management of mechanically ventilated patients with acute respiratory distress syndrome (ARDS) since its description by Ashbaugh et al. in 1967 [1]. As part of a lung protective ventilation strategy, PEEP improves ventilation distribution and potentially limits ventilator-induced lung injury (VILI) [2]. Although different methods to titrate PEEP based on oxygenation and respiratory mechanics have been evaluated in randomized clinical trials, the optimal strategy to improve clinical outcomes remains undefined [3].

The current European Society of Intensive Care Medicine (ESICM) guideline on ARDS does not make a recommendation on higher versus lower oxygenation-based PEEP or PEEP titration guided by respiratory mechanics compared to oxygenation [4]. In contrast, the American Thoracic Society (ATS) guideline on ARDS management suggests using higher rather than lower PEEP in patients with moderate to severe ARDS [5] based on two meta-analyses showing an association between higher PEEP and improved survival in this ARDS subpopulation [6, 7].

Management of severe ARDS patients with prone positioning (PP) and veno-venous extracorporeal membrane oxygenation (V-V ECMO), along with the associated changes in respiratory mechanics and ventilatory strategies, may also affect the physiological effects of PEEP [8–10]. In these situations, “optimal” PEEP may differ and warrant careful re-titration to balance potential benefits and harms.

This article reviews PEEP management in patients with severe ARDS, focusing on the physiological effects of personalized PEEP with respect to respiratory mechanics and cardiopulmonary function, and the implications of management with PP and V-V ECMO.

## PEEP in severe ARDS

### Physiological effects and rationale

Almost 50 years ago, Suter et al. described that “optimal” PEEP titrated to respiratory mechanics and oxygen delivery (DO_2_) could potentially improve cardiopulmonary function in patients with respiratory failure [11]. Since then, the primary goal for the use of PEEP has shifted from merely improving oxygenation to emphasizing lung protection, with a growing focus on the individual pattern of lung injury, lung and chest wall mechanics, and the hemodynamic consequences of PEEP [3, 4, 12].

**Fig. 1:**
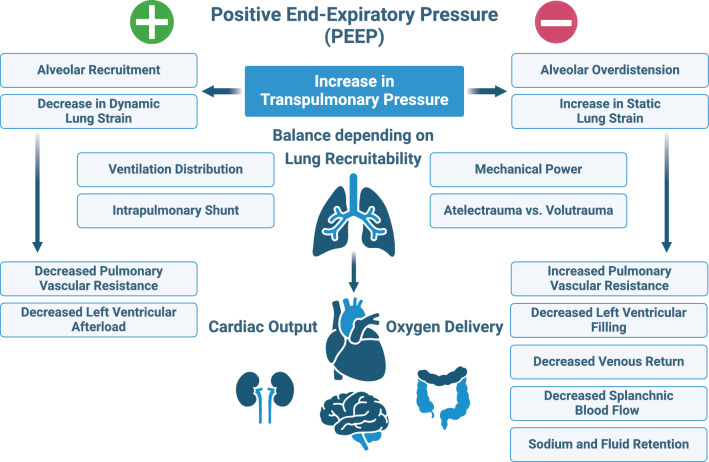
Cardiopulmonary effects of positive end-expiratory pressure

PEEP as part of a lung protective ventilation strategy can promote alveolar recruitment and limit atelectrauma, thereby reducing ventilation inhomogeneity and potentially preventing VILI [13] (Fig. 1). However, excessive PEEP may result in VILI due to alveolar overdistension and increased mechanical power (MP) transmission to the lung [14].

PEEP can adversely affect hemodynamics by decreasing the gradient for venous return, increasing pulmonary vascular resistance, and decreasing cardiac output [15] (Fig. 1). These hemodynamic consequences of excessive PEEP may increase the need for fluid administration and cardioactive drugs, which could impact patient outcomes [16, 17].

The balance between the benefits and harms of PEEP critically depends on its effect on lung recruitment, defined as reaeration of non- and poorly aerated lung parenchyma in response to increased airway pressures [18]. The application of PEEP increases transpulmonary pressure (P_TP_), i.e. the difference between airway and pleural pressure, in both non-dependent and dependent lung regions [19]. The resultant change in lung volume relative to the resting lung volume, termed functional residual capacity (FRC), represents lung strain. Lung strain is composed of static and dynamic components due to PEEP [end-expiratory lung volume (EELV) determined by PEEP relative to FRC] and tidal volume (V_T_) (V_T_ relative to EELV), respectively.

In cases of significant recruitability, PEEP-induced increases in P_TP_ lead to reaeration of lung parenchyma with minimal overdistension. While PEEP increases static strain, the corresponding increase in EELV significantly decreases dynamic strain with tidal ventilation, potentially improving gas exchange, lung protection, and limiting VILI [20, 21]. Conversely, excessive PEEP in cases of low recruitability fails to reaerate lung parenchyma significantly, increasing static strain without decreasing dynamic strain. This results in overdistension of aerated lung parenchyma, leading to increased lung stress and strain, and raising the risk for VILI as well as hemodynamic compromise (Fig. 1). It is important to note that due to the lung inhomogeneity in patients with ARDS, increasing P_TP_ with PEEP always involves a trade-off between reaeration and overdistension of aerated lung parenchyma [22], with "recruitability" indicating this balance. Although increasing PEEP may decrease lung inhomogeneity in patients with mild and moderate ARDS, significant recruitment or reduced lung inhomogeneity may not be achievable in severe ARDS patients [23] when airway plateau pressures are limited to the recommended 30 cmH_2_O [4].

In these cases, PP may be employed to enhance ventilation distribution, thereby reducing overdistension and cyclical alveolar opening and closing [24]. In severe ARDS patients with refractory gas exchange impairment or inability to provide lung protective ventilation, V-V ECMO facilitates gas exchange and allows for an “ultraprotective” ventilation strategy [25].

### Assessment and personalized PEEP strategy

Severe ARDS is characterized by a significant heterogeneity in the pattern of lung injury (focal vs. diffuse), lung and chest wall mechanics, hemodynamics, and potential multiorgan dysfunction [26, 27]. This variability, combined with the interaction of different ventilator strategies [28], complicates PEEP management and the definition of an optimal and personalized PEEP strategy [3, 4]. Although the optimal approach for managing PEEP in severe ARDS remains unclear, both the ESICM and ATS guidelines emphasize the importance of assessing the cardiopulmonary risk–benefit ratio when setting PEEP [4, 5].

**Fig. 2:**
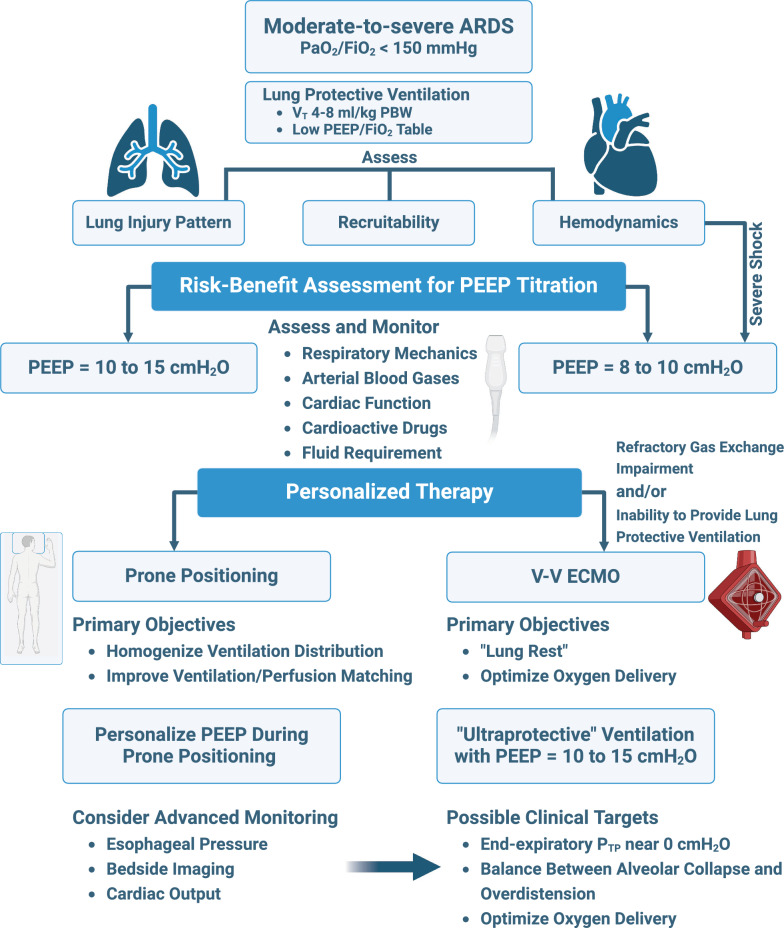
Possible algorithm for the risk–benefit assessment in PEEP titration and personalized therapy during prone positioning and V-V ECMO in patients with severe ARDS. PaO_2_/FiO_2_, arterial partial pressure of oxygen divided by the fraction of inspired oxygen; V_T_, tidal volume; PBW, predicted body weight; PEEP, positive end-expiratory pressure; V-V ECMO, veno-venous extracorporeal membrane oxygenation; P_TP_, transpulmonary pressure

Assessing lung recruitability to estimate the theoretical response to higher PEEP can be done using physiological parameters (e.g. oxygenation, ΔP, P_TP_) [29–31], bedside maneuvers (e.g. recruitment-to-inflation ratio) [32], and imaging techniques (e.g. computed tomography, electrical impedance tomography, ultrasound) [33–35]. However, PEEP management based solely on recruitability may not provide a truly personalized approach to ventilator management in severe ARDS patients [36]. A more comprehensive approach involves evaluating the interaction between PEEP, treatment strategies (PP, “ultraprotective” ventilation with very-low V_T_), resulting gas exchange and P_TP_, as well as considering the “biological” costs related to hemodynamic variables and fluid requirements (Fig. 2).

The competing effects of PEEP in balancing lung protection and hemodynamic stability were highlighted in a reanalysis of the EPVent-2- trial. This study showed that the effect of PEEP strategy on mortality depended on the severity of multiorgan dysfunction [37]. PEEP titrated to end-expiratory P_TP_ near 0 cmH_2_O was associated with improved survival compared to more positive or negative values [37], indicating the need to balance atelectrauma and overdistension [22]. At the bedside, this approach may be combined with non-invasive methods, such as electrical impedance tomography (EIT), to visualize ventilation distribution and estimate the response to PEEP semi-quantitatively [38, 39].

Given the high prevalence of hemodynamic impairment, including acute *cor pulmonale*, in severe ARDS patients [40], the effect of PEEP on hemodynamic variables and the need for cardioactive drugs and fluids should be considered. Monitoring the interaction between mechanical ventilation and cardiopulmonary function using echocardiography and advanced hemodynamic monitoring, such as pulmonary artery catheter and transpulmonary thermodilution, is recommended in this ARDS subgroup [15]. In the presence of right ventricular failure, which is associated with increased mortality [40], the response to PEEP concerning P_TP_, alveolar recruitment and overdistension, as well as the effects on cardiopulmonary function, should be periodically reassessed [41].

Management with PP and V-V ECMO may improve right ventricular function and optimize hemodynamics in severe ARDS patients [15, 42, 43]. These strategies can enhance oxygenation, homogenize regional P_TP,_ and potentially reduce the intensity of ventilation.

### Clinical implications

- In the absence of an “optimal” PEEP, which simultaneously enhances alveolar recruitment and gas exchange while avoiding overdistension and hemodynamic impairment, a moderate level of PEEP that provides lung protection while minimizing hemodynamic compromise may be acceptable.
- Once PEEP is set, it is crucial to periodically reassess its effects on cardiopulmonary function.

## Prone positioning

### Physiological effects of prone positioning and interaction with PEEP

Current guidelines [4, 5] recommend PP for patients with moderate-to-severe ARDS (PaO_2_/FiO_2_ < 150 mmHg) to reduce mortality based on findings from the PROSEVA trial [44]. In the most severe ARDS patients with PaO_2_/FiO_2_ < 80 mmHg, both PP and V-V ECMO are associated with improved outcomes relative to low or moderate V_T_ alone, according to a recent meta-analysis by Sud et al. [45]. Given that PP is a critical treatment for severe ARDS [46], understanding the physiological changes that occur during PP is essential for optimizing PEEP management. PP and PEEP can interact synergistically to influence respiratory mechanics, lung volumes, and gas exchange (Table 1).

**Table 1:** Implications of prone positioning for PEEP management in patients with severe ARDS

Physiological effects of prone positioning	Implications for PEEP management
Reduced pleural pressure gradient	**Improved ventilation homogeneity:** PP can enhance ventilation distribution across different lung regions, potentially allowing for a wider range of effective PEEP levels. This can result in better compliance for both non-dependent and dependent lung regions at the same PEEP setting
Increased chest wall elastance	**Possible reduction in alveolar overdistension:** PP, combined with higher PEEP levels, may help mitigate alveolar overdistension by promoting a more uniform distribution of ventilation
Improved ventilation homogeneity	**Reduced dependence on higher PEEP:** With improved ventilation homogeneity from PP, there may be less need for excessively high PEEP levels to achieve homogeneous ventilation across the lung
Lung recruitment*	**Synergistic effects:** The combination of PP and moderate PEEP may produce synergistic effects, potentially allowing for a reduction in PEEP levels while preserving EELV and optimizing gas exchange
Improved ventilation/perfusion matching	**Reduced dependence on PEEP for oxygenation:** PP can enhance gas exchange efficiency, potentially decreasing the reliance on higher PEEP to improve oxygenation
Right ventricular unloading*	**Potential for moderate PEEP:** PP may facilitate the use of moderate PEEP levels, optimizing both ventilation and hemodynamics while minimizing potential adverse effects associated with high PEEP

PEEP, positive end-expiratory pressure; ARDS, acute respiratory distress syndrome; PP, prone positioning; EELV, end-expiratory lung volume

^*^Individual responses to prone positioning can vary, and effects such as lung recruitment and right ventricular unloading may be influenced by patient-specific factors

In the supine position, gravity causes a vertical pleural pressure gradient that reduces P_TP_ from ventral to dorsal lung regions [8]. This pleural pressure gradient is exacerbated in severe ARDS patients by the pressure of the edematous lung, sedation, and neuromuscular blockade [47, 48], leading to collapse of dorsobasal alveoli and ventilation inhomogeneity [49]. Experimental data suggest an increased pleural pressure gradient with lower PEEP levels, resulting in predominant ventilation of non-dependent lung regions in the supine position [8]. While increasing PEEP can promote dorsobasal alveolar recruitment, it may also cause overdistension of non-dependent lung regions [50]. PP alleviates the pleural pressure gradient by reducing the compressive force of the mediastinum, resulting in a more uniform distribution of regional end-expiratory and end-inspiratory P_TP_ and thus ventilation [8]. Furthermore, PP increases EELV through lung recruitment associated with an uniform distribution of P_TP_ [51]. This homogenization of ventilation distribution and lung recruitment reduces ventilation/perfusion mismatch and shunt, thereby improving gas exchange in ARDS patients during PP [24].

As mentioned earlier, PEEP alone may not induce significant lung recruitment in severe ARDS patients and could lead to overdistension of aerated lung parenchyma [23]. Experimental data suggest that the synergistic effects of PEEP and PP optimize regional compliance and minimize transpulmonary driving pressure (ΔP_TP_) in both dependent and non-dependent lung regions [8]. Therefore, by reducing the pleural pressure gradient, the combination of PEEP and PP facilitates recruitment of dependent lung regions without overdistending non-dependent lung regions, potentially mitigating VILI.

PP enhances lung recruitment and decreases alveolar instability and overdistension observed at high PEEP levels in ARDS patients [50]. Another important mechanism during PP is the increase in chest wall elastance [52, 53], which modifies regional P_TP_ and induces recruitment by shifting lung aeration dorsally, thus improving ventilation homogeneity [54]. This improvement in ventilation/perfusion matching and reduced shunt improves gas exchange [55–57], while homogenized regional P_TP_ may mitigate the cardiopulmonary effects of mechanical ventilation [15]. In severe ARDS patients with right ventricular failure, PP may help unload the right ventricle and optimize hemodynamics, potentially contributing to the improvement in patient outcomes [42, 43].

The dorsal ventilation shift and alveolar recruitment during PP may decrease ΔP_TP_, a surrogate for true lung stress independent of chest wall mechanics [8, 53]. Notably, experimental and clinical data recommend that minimal ΔP_TP_ can be achieved with lower PEEP during PP compared to supine positioning [8, 58], suggesting recruitment of previously non-aerated lung units and/or improved mechanical properties of already ventilated lung units [59]. The optimal PEEP level during PP and whether it should be adjusted after changes in patient positioning remain debated in the absence of studies with patient-centered outcomes [60]. This challenge is compounded by the fact that ventilator-based measurements do not always correlate with changes in the lung-to-respiratory system elastance ratio, which significantly alters lung energy transfer during PP [61–63].

### Clinical data

In the PROSEVA trial, which demonstrated a reduction in mortality with PP, patients were initially ventilated with a PEEP of approximately 10 cmH_2_O according to the lower PEEP/FiO_2_ table [44]. PEEP was gradually decreased to 8–9 cmH_2_O over the following seven days, without adjustment based on patient positioning [44]. Improved lung protection due to more uniform distribution of lung stress and strain during the respiratory cycle has been cited as a major factor contributing to improved outcomes in the PROSEVA trial [24, 50, 64]. Although a physiological study using the PROSEVA protocol did not reduce driving pressure (ΔP) or MP with PP, it did show increased EELV and decreased ΔP_TP_, suggesting more even distribution of lung stress and strain during PP [53]. Furthermore, PP may allow for reduced elastic power transfer per aerated lung volume, as comparable P_TP_ and EELV can be achieved at lower airway pressures compared to supine positioning [58].

A recent physiological study by Morais et al. demonstrated the necessity of individualizing PEEP based on patient positioning due to significant changes in respiratory mechanics during PP [65]. Using EIT and esophageal manometry, the authors found a heterogeneous response in regional and global respiratory mechanics during PP, requiring PEEP adjustment of PEEP of at least 4 cmH_2_O in approximately 50% of patients [65]. Conversely, Mezidi et al. found no significant changes in PEEP adjusted to physiologic endpoints between supine positioning and PP [66]. In another recent study, PEEP was titrated based on the lowest static respiratory system compliance or positive end-expiratory P_TP_ with respect to FiO_2_ [67] in both supine positioning and PP [53]. With both strategies, PEEP titration according to physiologic targets was lower during PP, resulting in reduced airway pressures and MP, while preserving EELV and improving oxygenation and hemodynamics. Esophageal pressure-guided ventilation targeting an end-expiratory P_TP_ of 0 to 2 cmH_2_O [37] led to a median PEEP reduction of 5 cmH_2_O during PP compared to supine positioning, demonstrating lower elastic power transfer, improved gas exchange, and DO_2_ [58].

### Implications of prone positioning for PEEP management

- The potential synergy between PP and PEEP may help to homogenize ventilation, recruit previously non-aerated lung units, and/or enhance the mechanical properties of already ventilated lung units without causing regional overdistension.
- PP may enable a reduction in PEEP, thereby reducing the energy required per aerated lung volume while preserving EELV, optimizing gas exchange, and maintaining hemodynamic stability.
- The significant variability in individual responses to PP, including changes in respiratory mechanics, P_TP_, and gas exchange, underscores the need for periodic reevaluation of PEEP settings during PP.

## Veno-venous extracorporeal membrane oxygenation

### Rationale and potential indications for V-V ECMO

In patients with the most severe ARDS and refractory gas exchange impairment, defined by eligibility criteria from the EOLIA trial (PaO_2_/FiO_2_ < 50 mmHg for > 3 h, or a PaO_2_/FiO_2_ of < 80 mmHg for > 6 h, or a pH of < 7.25 with a PaCO_2_ of ≥ 60 mmHg for > 6 h, with the respiratory rate increased to 35 breaths/min and mechanical ventilation settings adjusted to keep a plateau pressure of ≤ 32 cmH_2_O [68]), V-V ECMO is recommended according to current guidelines [4, 5]. The extracorporeal circuit facilitates gas exchange while employing an "ultraprotective” ventilation strategy aimed at achieving "lung rest" [10, 69]. This strategy is adopted by most medium-to-high volume ECMO centers and aims to reduce lung stress and strain by decreasing V_T_, respiratory rate, and airway pressures, thereby minimizing energy transfer to the inflamed lung parenchyma [70]. Although correcting refractory hypoxemia is critical, the primary benefit of managing severe ARDS patients with V-V ECMO is thought to be the reduced risk of VILI associated with less intensive mechanical ventilation [25].

Given that the extracorporeal circuit ensures adequate gas exchange, two primary objectives for ventilator management in severe ARDS patients undergoing V-V ECMO emerge: (1) Lung protection to minimize VILI; and (2) Reduction of the hemodynamic impact of mechanical ventilation to optimize DO_2_ (Table 2). The latter is particularly crucial for severe ARDS patients with right ventricular failure unresponsive to conservative treatments. In such cases, V-V ECMO can facilitate gas exchange while allowing for an "ultraprotective" ventilation strategy [15] that minimizes ventilatory settings and associated hemodynamic effects. This approach has been demonstrated to improve right ventricular function by reducing hypoxic pulmonary vasoconstriction and intrathoracic pressures, which lowers right ventricular afterload and enhances right ventricular coupling [71, 72].

**Table 2:** Implications of V-V ECMO treatment for PEEP management in patients with severe ARDS

Implications of V-V ECMO treatment	Implications for PEEP management
“Lung rest” strategy	**Minimize VILI:** Moderate PEEP levels to reduce mechanical power transmission. Inadequate low PEEP can lead to progressive alveolar collapse and increased pulmonary vascular resistance*
“Ultraprotective” tidal volume	**Reduced transpulmonary pressure:** Possible interaction with cyclic alveolar opening and closing (atelectrauma)
Very low lung volume due to severe disease	**Limit end-tidal overdistension:** Necessity of high airway pressures for significant recruitment*
High ventilation inhomogeneity	**Highly variable recruitability:** Possible paradoxical effects with higher PEEP levels*
Gas exchange primarily through V-V ECMO	**Extracorporeal gas exchange:** Reduced dependence on PEEP to ensure oxygenation
Right ventricular unloading*	**Potential for moderate PEEP:** Moderate PEEP to target lower airway pressures during acute *cor pulmonale*

V-V ECMO, veno-venous extracorporeal membrane oxygenation; PEEP, positive end-expiratory pressure; ARDS, acute respiratory distress syndrome; VILI, ventilator-induced lung injury

^*^ Individual responses to “ultraprotective” ventilation can vary, and effects such as lung recruitment and right ventricular unloading may be influenced by patient-specific factors

### PEEP as part of an ultraprotective ventilation strategy

The “ultraprotective” ventilation strategy facilitated by the extracorporeal circuit has important implications for PEEP management. Firstly, there is no reliance on PEEP to ensure oxygenation through alveolar recruitment, as gas exchange is primarily managed by the extracorporeal circuit [70, 73]. Secondly, the reduction in V_T_ and respiratory rate during "ultraprotective" ventilation alters the role of adequate PEEP in increasing EELV and limiting dynamic strain during tidal ventilation. Moreover, the importance of sufficient PEEP to stabilize edematous alveoli and prevent cyclic alveolar collapse and reopening needs reevaluation in these patients [73].

While the reduction in V_T_ during "ultraprotective" ventilation lowers the risk of overdistension, a V_T_ of 2 to 4 ml/kg predicted body weight, combined with moderate PEEP levels and limited airway plateau pressures, may still lead to regional overdistension and VILI in patients with severe ARDS, particularly those with very low EELV [74, 75]. According to the current ARDS guidelines and using a PEEP/FiO_2_ table, patients eligible for V-V ECMO should initially be managed with an empirical PEEP of 16 to 24 cmH_2_O [4, 5]. The Extracorporeal Life Support Organization (ELSO) recommends PEEP levels between 10 and 24 cmH_2_O while maintaining an inspiratory plateau pressure lower than 25 cmH_2_O [70]. Consequently, PEEP management during V-V ECMO treatment shows considerable variation between centers [76], with participating centers in the LIFEGARDS study using a mean PEEP of 11 ± 3 cmH_2_O [10]. Similarly, the CESAR and EOLIA trials utilized a comparable PEEP range of 10 to 12 cmH_2_O to facilitate "lung rest" [68, 77]. Despite numerous studies evaluating the impact of ventilator settings, including PEEP, on patient outcomes during V-V ECMO [10, 76, 78, 79], findings regarding optimal PEEP have been inconsistent. Recent analyses identified more integrative ventilatory parameters such as ΔP and MP as independent predictors of mortality in this ARDS subgroup [80, 81]. To date, the optimal PEEP strategy to improve outcomes in severe ARDS patients managed with V-V ECMO remains unknown. Following the initiation of extracorporeal support and "ultraprotective" ventilation, using a moderate level of PEEP (10 to 15 cmH_2_O) may be reasonable to balance lung protection and hemodynamic stability in this ARDS subgroup. Further personalization of PEEP settings should then be guided by clinical assessment of the benefits and risks of PEEP in terms of cardiopulmonary function to minimize VILI, maintain hemodynamic stability, and optimize DO_2_ (Fig. 1).

### Clinical and experimental data

In patients with the most severe ARDS requiring V-V ECMO treatment, recruitability varies widely [82], influencing the benefits and risks associated with PEEP. In a physiological study using EIT, Franchineau et al. identified an optimal PEEP of 15 cmH_2_O in 47% and 10 cmH_2_O in 40% of the included patients, defined as a compromise between preventing alveolar collapse and avoiding overdistension, which can lead to atelectrauma and volutrauma, respectively [83]. Graf et al., quantifying lung volumes via computed tomography in V-V ECMO patients, found that titrated PEEP increased static lung strain approximately 1.5-fold without recruiting dependent lung regions, resulting in significantly higher non-dependent lung strain [84]. Despite the moderate-to-high mean PEEP of 15.4 cmH_2_O, tidal recruitment was observed with a V_T_ of 3 ml/kg predicted body weight. During V-V ECMO and "ultraprotective" ventilation, setting PEEP to minimize ΔP and prevent cyclic alveolar collapse while limiting airway plateau pressures to avoid overdistension in non-dependent lungs may prove challenging [23]. Accepting cyclic alveolar opening and closing (atelectrauma) while avoiding high airway pressures linked to volutrauma, mainly in non-dependent lungs, may be preferable for reducing pulmonary inflammation during the initial phases of V-V ECMO [85]. This approach minimizes energy transfer to lung parenchyma, which is reflected in MP [86]. Despite the ongoing discussions regarding the significance of each MP component, high overall MP increases the risk of VILI in patients with ARDS [14] and has been associated with mortality during V-V ECMO treatment [81].

Adopting a less invasive ventilatory strategy with moderate levels of PEEP to promote “lung rest” may provide a favorable balance between lung protection and cardiopulmonary function. In an experimental ARDS model managed with V-V ECMO, a moderate PEEP level of 10 cmH_2_O minimized lung injury while maintaining hemodynamic stability. Conversely, inadequate PEEP, whether too low or high, exacerbated lung injury or precipitated hemodynamic collapse, respectively [87]. Experimental studies comparing equivalent total MP with differing static and dynamic components revealed similar lung injury outcomes, with high PEEP exerting the greatest impact on hemodynamics and necessitating fluid administration [88]. However, inadequate PEEP during V-V ECMO treatment can lead to progressive alveolar collapse, which may subsequently increase pulmonary vascular resistance [89, 90]. Another aspect to consider when using moderate PEEP levels during “ultraprotective” ventilation in the most severe ARDS patients is the presence of complete airway closure, which may confound assessments of respiratory mechanics and contribute to atelectasis due to denitrogenation [91, 92].

Changes in regional P_TP_ in response to PEEP determine the cardiopulmonary effects of mechanical ventilation, particularly in severe ARDS patients with marked ventilation heterogeneity [15]. Atelectasis due to insufficient PEEP can lead to right ventricular failure [89, 90], while lung overdistension due to excessive PEEP impairs pulmonary circulation, and reduces cardiac output [15]. Individual recruitability and the balance between lung reaeration and overdistension are therefore critical considerations in PEEP management to maintain hemodynamic stability and optimize DO_2_. A recent study in severe ARDS patients managed with V-V ECMO compared a moderate empirical PEEP level of 10 cmH_2_O, the lowest PEEP recommended by the ELSO, with individualized PEEP titration based on highest respiratory system compliance (mean 16.2 ± 4.7 cmH_2_O) and end-expiratory P_TP_ of 0 cmH_2_O (mean 17.3 ± 4.7 cmH_2_O) during "ultraprotective" ventilation [93]. Although respiratory mechanics-guided PEEP titration decreased ΔP_TP_ compared to empirical PEEP of 10 cmH_2_O, both approaches increased MP and lung stress, leading to reduced cardiac output and DO_2_, potentially compromising the extracorporeal circuit's goal of maintaining tissue normoxia. Notably, lung recruitability was not formally assessed using imaging or physiological maneuvers [32, 34], and higher PEEP did not significantly recruit lungs in the study cohort [93], consistent with previous findings in this ARDS subgroup [23]. The high disease severity and multiorgan dysfunction in the study cohort may have also contributed to the hemodynamic impairment associated with higher PEEP [37].

### Implications of V-V ECMO for PEEP management

- Setting PEEP during “ultraprotective” ventilation in severe ARDS patients managed with V-V ECMO, especially during the initial phases of treatment involving multiorgan dysfunction and shock, should strive to balance lung protection with hemodynamic stability.
- A moderate level of PEEP (10 to 15 cmH_2_O), as part of a “lung rest” strategy, may be appropriate for this ARDS subgroup to minimize VILI, maintain hemodynamic stability, and optimize DO_2_.
- Further personalization of PEEP during V-V ECMO requires periodic assessment of the benefits and risks associated with PEEP in terms of cardiopulmonary function.

## Conclusions

In patients with severe ARDS characterized by a significant heterogeneity in lung injury patterns, respiratory mechanics, hemodynamics, and potential multiorgan dysfunction, careful assessment of the benefits and risks associated with PEEP in terms of cardiopulmonary function is warranted. In these patients, the use of PP and its synergism with PEEP to homogenize ventilation distribution has important implication for PEEP management, necessitating reevaluation of PEEP settings during PP. Management of the most severe ARDS patients with V-V ECMO facilitates gas exchange and allows for a “lung rest” strategy. A moderate level of PEEP during “ultraprotective” ventilation may balance lung protection and hemodynamic stability in this ARDS subgroup. Individualized assessment and careful PEEP titration are crucial for optimizing ventilator management and improving patient outcomes.

## Data Availability

Not applicable.

## Abbreviations

ΔP: Driving pressure
ΔP_TP_: Transpulmonary driving pressure
ARDS: Acute respiratory distress syndrome
ATS: American Thoracic Society
DO_2_: Oxygen delivery
EELV: End-expiratory lung volume
EIT: Electrical impedance tomography
ELSO: Extracorporeal Life Support Organization
ESICM: European Society of Intensive Care Medicine
FRC: Functional residual capacity
MP: Mechanical power
PaCO_2_: Arterial partial pressure of carbon dioxide
PaO_2_/FiO_2_: Arterial partial pressure of oxygen divided by the fraction of inspired oxygen
PEEP: Positive end-expiratory pressure
PP: Prone positioning
P_TP_: Transpulmonary pressure
VILI: Ventilator-induced lung injury
V_T_: Tidal volume
V-V ECMO: Veno-venous extracorporeal membrane oxygenation
